# Genetic Variants Linked with the Concentration of Sex Hormone-Binding Globulin Correlate with Uterine Fibroid Risk

**DOI:** 10.3390/life15071150

**Published:** 2025-07-21

**Authors:** Marina Ponomarenko, Evgeny Reshetnikov, Maria Churnosova, Inna Aristova, Maria Abramova, Vitaly Novakov, Vladimir Churnosov, Alexey Polonikov, Denis Plotnikov, Mikhail Churnosov, Irina Ponomarenko

**Affiliations:** 1 Department of Medical Biological Disciplines, Belgorod State National Research University, 308015 Belgorod, Russia; 1256888@bsuedu.ru (M.P.); reshetnikov@bsuedu.ru (E.R.); churnosovamary@gmail.com (M.C.); aristova@bsuedu.ru (I.A.); abramova_myu@bsuedu.ru (M.A.); 659864@bsuedu.ru (V.N.); 958561@bsuedu.ru (V.C.); polonikov@rambler.ru (A.P.); ponomarenko_i@bsuedu.ru (I.P.); 2 Department of Biology, Medical Genetics and Ecology, Kursk State Medical University, 305041 Kursk, Russia; 3 Research Institute for Genetic and Molecular Epidemiology, Kursk State Medical University, 305041 Kursk, Russia; 4 Genetic Epidemiology Lab, Kazan State Medical University, 420012 Kazan, Russia

**Keywords:** association, sex hormone-binding globulin, SNP, uterine fibroids

## Abstract

In this study we searched for correlations between polymorphic variants that determine sex hormone-binding globulin concentration (SHBG_con_) and uterine fibroids (UFs). The work was performed on a sample of 1542 women (569 with UFs and 973 without UFs [control]), from whom we obtained experimental data on the distribution of nine single-nucleotide polymorphisms (SNPs) affecting the SHBG_con_ (data confirmed in genome-wide association studies [GWASs]). When searching for associations with UFs, both the independent effects of SNPs and the effects of their SNP–SNP interactions (SNP-SNP_ints_) were taken into account during the “deep study” of the functionality of seven important UF loci and 115 strongly linked [r^2^ ≥ 0.80] variants (an in silico methodology was used). As the results show, two SHBG_con_-related SNPs correlated with UF risk: rs3779195 [T/A] *BAIAP2L1* (OR_AA_ = 0.38; 95%CI_AA_ = 0.20–0.91; p_perm(AA)_ = 0.023) and rs440837 [A/G] *ZBTB10* (OR_GG_ = 1.93; 95%CI_GG_ = 1.17–3.14; p_perm(GG)_ = 0.010). At the same time, seven SHBG_con_-related SNPs interacting with each other (four models of such SNP-SNP_ints_ [p_perm_ ≤ 0.01)] were found to influence UF risk. These SHBG_con_-related SNPs, determining susceptibility to UF, showed strong functional relevance and were involved in pathways of gene transcription regulation, interactions with hormone ligand-binding receptors, the content control of SHBG, testosterone, liver enzymes, lipids, etc. This study’s results demonstrate the effect of significant SHBG_con_-related genetic determinants of UF risk.

## 1. Introduction

Uterine fibroids (UFs) are the most common pelvic tumors, affecting about 70% of women worldwide [1]. The disease is clinically manifested in at least 25–50% of patients [2,3]. The tumor reveals itself by severe menstrual bleeding, often leading to severe iron deficiency anemia, pelvic pain, and reproductive disorders, including infertility and pregnancy complications [1,3]. UFs often require surgical treatment [4,5]. UFs are the main cause of hysterectomies, accounting for at least one third of all hysterectomies [2]. The high UF prevalence has a significant economic impact on healthcare systems worldwide. Treatment costs for UF patients amount to hundreds of millions of dollars in countries such as Germany (USD 348 million) and France (USD 120 million) and reach tens of billions of dollars in the USA (USD 34.4 billion) [6]. It should be emphasized that the cost of treating women with UFs exceeds the cost of treating women with such common tumors as breast cancer and ovarian cancer [6]. In addition to direct healthcare costs, indirect costs due to temporary disability and the disability of women with uterine fibroids are estimated at USD 1.6–17.2 billion annually worldwide [7].

UFs are a genetically determined disorder, with a substantial hereditary contribution to UF development (26–69%, European twin studies data) [8,9]. The genetic determinants of UFs have been identified in more than ten GWASs (several dozen UF risk loci were detected, including genes “involved” in sex hormone pathways [*ESR1*, *FSHB*, etc.]) [10,11]; however, only a small part of UF heredity (≈13%) can be explained by the known GWAS (SNP) data [11], which confirms the relevance of further UF genetic studies aimed at detecting as-yet-unknown genetic risk factors for UFs.

The effect of sex hormones (such as estrogens and testosterone) on UFs is beyond doubt [12,13]. Estrogens are believed to stimulate the growth of myomatous nodes (interacting with estrogen α receptors and enhancing the expression of progesterone receptors, they increase the proliferative activity of uterine smooth muscle cells, etc.) [13]. The effect of testosterone on UF development is also significant (local transformation into estrogens under the action of aromatases, proapoptotic/antiproliferative effects, etc.); however, there is currently no definitive opinion in the literature on the direction of the effect of testosterone (risky/protective) on UF risk [12,13,14]. The pathophysiological effects of these hormones in the body (including UFs) will largely depend on SHBG [14]). SHBG, binding and transporting a major portion of testosterone (80%) [12] and estrogens (38%) [15], thus ensures the “regulation” of the level of the free fractions of these sex hormones in the organism (they account for 2% of estrogens and 1% of testosterone), which exhibit biological activity in the female (i.e., they are bioactive) [12,15]. Thus, the above data suggest that the modulation of testosterone and estrogen concentrations in the organism caused by SHBG (due to binding/release) (their low concentrations at high levels of SHBG and, conversely, high concentrations of testosterone/estrogen at low levels of SHBG) will be important in UF pathology. So, based on these literature materials, it can be noted that the main hypothetical biological mechanism by which SHBG levels can influence UF development is their significant effect on concentrations of bioavailable androgens/estrogens in the organism, which will be important in the UF formation.

SHBG_con_ in the organism is genetically determined, as demonstrated by numerous GWASs [16,17,18,19,20,21,22]. However, the role of polymorphisms/genes that determine SHBG_con_ in UF development remains unexplored at the moment. To date, only one study by Wang et al. has investigated this association using Mendelian randomization (MR) of two cohorts, FinnGen and FibroGENE. While no significant association between genetic variants of SHBG_con_ and the risk of UF was found in the meta-analysis of the two studies, a cohort-specific association was identified in FibroGENE [23]. There are no other experimental genetic studies on this issue. So, the lack of knowledge on the role of the genetic determinants of SHBG_con_ in UF development dictates the need for research in this area, which is the subject of our study. The aim of the work is to examine correlations between polymorphic variations that determine the SHBG_con_ and UF.

## 2. Materials and Methods

### 2.1. Study Subjects

The phasing/design/ethical aspects of this study were reviewed and supported by the Ethics Committee (Medical Research) of Belgorod State National Research University. Each woman surveyed gave her consent (signed personally) to participate in the study. The overall number of the genetically studied group of women was *n* = 1542, including 569 with UF and 973 without UF [control]. All subjects were born in central Russia and were Europeans (Russian nationality) [24,25,26]. The UF diagnosis in all patients (ICD-10:code D25) was confirmed morphologically after hysterectomy in the gynecological department of the Belgorod Perinatal Center [27]. All women in the control group underwent a comprehensive medical evaluation (conducted at the Belgorod Perinatal Center), including a clinical examination, a medical history review, and pelvic ultrasound screening, specifically evaluated for proliferative disorders (UFs, endometriosis/adenomyosis, and endometrial hyperplasia). The control group included women who had no clinical, anamnestic, or ultrasound signs of proliferative diseases of the pelvic organs [27]. Despite the fact that ultrasound examination, used in the work to confirm the absence of UF in the control group, is the “gold standard” of uterine imaging and serves as the main method of diagnosing UFs, there is a possibility of non-detection of UFs (for example, small-sized UFs, cervical UFs, etc.) when it is used in the examination of women in the control group, which may be a limitation of the present study. Women with malignant diseases of the pelvic organs/breast, the severe pathology of the immune/vital system/organs were excluded from the study. The main characteristics of the UF/UF-free studied groups are shown in Table 1 (these materials were submitted in the previous genetic study of UFs [27]). The data in Table 1 point out the “UF vs. UF-free” differences—in terms of such indicators as age, weight, BMI, family history, a history of infertility, artificial abortions (and their number), chronic endometritis—which formed the basis for using these characteristics in “UF-SHBG_con_-related SNP” association calculations as confounders.

### 2.2. Experimental DNA Study (Selection/Genotyping of SHBG_con_-Related Genetic Variants)

For the experimental study, DNA samples (isolated from peripheral blood using phenol, chloroform, and ethanol [28]) obtained during a previously conducted genetic study of UFs were used [27]. The DNA samples were stored in low-temperature (−80 °C) refrigerators (kelvinators), and had the required degree of purity (“260 nm/280 nm” ratio = 1.7–2.0) [29] and concentration (10–20 ng/mL) (the data on the DNA density/concentration were obtained on a NanoDrop™ 2000 (Thermo Fisher Scientific Inc., Waltham, MA, USA) [30]). Nine polymorphisms that showed associations with SHBG_con_ in previously performed GWASs were included in the work ([16,18,20,21,22]; detailed GWAS data on associations of the studied SNP with SHBG_con_ are presented in Table 2 and Supplementary Table S1). Also, when selecting polymorphisms, their potential functionality was taken into account (the assessment of the potential functionality of SNPs was carried out in silico using the bioinformatic online resource Haploreg—accessed 24 November 2024 [31]; the data obtained are presented in Supplementary Table S2). These are polymorphisms rs3779195 [T/A] *BAIAP2L1*, rs17496332 [A/G] *PRMT6*, rs8023580 [T/C] *NR2F2*, rs780093 [C/T] *GCKR*, rs440837 [A/G] *ZBTB10*, rs4149056 [T/C] *SLCO1B1*, rs10454142 [T/C] *PPP1R21*, rs12150660 [G/T] *SHBG*, and rs7910927 [G/T] *JMJD1C*. The SNP genotyping procedure was performed on CFX96 amplifiers [32]. The method of “blind” SNPs re-genotyping (for this purpose, we conducted repeated analyses for approximately 5% of the DNA samples [33]), which we employed to verify the accuracy of the genetic data, demonstrated a perfect match in the outcomes (specific genotypes) for 99% of the “blind” re-genotyped SNPs/samples, confirming the satisfactory quality of the genetic analysis.

### 2.3. Statistical Analysis (SNP–Multi-SNP Association Examined)

Previously, before analyzing the UF-SNP associations, the implementation of the Hardy–Weinberg (HW) rule by all SNPs among the UF/UF-free groups was considered [34,35].

We searched for an association of the SHBG_con_-related polymorphisms with UFs in two directions [36]: (a) the relationship of individual loci with UFs was studied; (b) the contribution of SNP-SNP_int_ (multi-SNP association) to exposure to UF was considered.

The association rates of individual SNPs with UFs (such as OR, 95%CI) [37]) were calculated using the logistic regression (the four most commonly used genetic-statistical models were used to evaluate various types of allelic variant interactions: dominant/additive/recessive/allelic [38]). The calculations were performed in gPLINK (a Java-related program [v.2.050]) [39] and took into account the effects of covariates (as indicated above, Table 1, these were age, weight, BMI, family history, a history of infertility, artificial abortions (and their number), and chronic endometritis), multiple comparisons (a permutation test was performed [adaptive version]) [40]) and the power of the associative connections (the Quanto software [v.1.2.4] was employed [41]). The values of “p_perm_” below 0.05 and “powers” above 80% were the basis for identifying reliable “UF-SNP” associations [42].

The MB-MDR (R-integrated) [43], GMDR (Java-integrated) [44,45], and MDR (Java-integrated) [46] programs were used to search/analyze the most UF-significant SNP-SNP_int_. When preparing data files for calculations in the MB-MDR, GMDR, and MDR programs, the missing genotype values were imputed using the MDR-Data Tool Software Overview (Java-integrated, v.3.0.2). All the UF-significant models we obtained took into account the necessary covariates (which are listed above) and were confirmed by permutation testing [40]. The permutation test was demonstrated to be efficient for the analysis of large massifs of GWAS data without reducing power [40]. As the most UF-significant SNP-SNP_int_ models, we considered models that met the following requirements: (1) the level of their statistical significance after the 1st stage of MB-MDR analysis corresponded to a special threshold level “p_border_” (determined based on the correction of the standard value “*p* = 0.05” by the number of genotype combinations considered at different SNP-SNP_int_ levels/loci [i.e., the Bonferroni correction was introduced]), which corresponded to the values of p_border_ = 1.39 × 10^−3^ (0.05/36), p_border_ = 5.95 × 10^−4^ (0.05/84), and p_border_ = 3.97 × 10^−4^ (0.05/126) for SNP-SNP_int_ models with 2, 3, and 4 loci, respectively [34]; (2) the level of their statistical significance after the 2nd stage of the MB-MDR analysis (the models were validated by permutation testing to minimize false positive results; 1000 permutations were performed) corresponded to the values of “p_perm_” < 0.01 (2- and 3-locus models) and < 0.001 (4-locus models). The two criteria mentioned above for identifying the most UF-significant SNP-SNP_int_ models were met only by models of the 3rd and 4th levels (two models of each level), which were included in our work.

Next, we performed a cross-validation of these four most UF-significant SNP-SNP_int_ models using the GMDR method/program (Java-integrated) [44,45]. The indicators of the cross validation consistency (CVC), testing balanced accuracy (TBA), sensitivity (S_e_), and specificity (S_p_) of the models were calculated, taking into account the correction for necessary covariates. A correction for multiple comparisons was performed using a permutation test in Perl script (“perl GMDR_permutatin.pl”) GMDR software (v.1.0). In total, 1000 permutations (with 10 cross-validations) were performed, which ensures the level of statistical significance of the validated model for samples of more than 1000 individuals with at least p_perm_ < 0.001.

The results derived by us in the study of “UF-SNP” associations were visualized (in the form of the SNP/SNP-SNP_int_ contribution to the UF entropy [47]) on a graph (using the MDR program [46]).

### 2.4. The Evaluation of the Possible Functionality of UF-Correlated Variants: An In Silico Study

Following the identification the UF-associated loci, we performed the functional annotation of these variants and their LD proxies (r^2^ value: 0.80–1.00 [48,49]) to investigate the biological mechanisms of the observed association between SHBG_con_-related SNPs and UF. To address this challenge, we used the following genomic/bioinformatic online resources [50,51]: (1) HaploReg_accessed_24 November 2024 [31]; (2) GTExportal_accessed_25 December 2024 [52]; and (3) STRING_accessed_08 December 2024 [53].

## 3. Results

All analyzed loci demonstrated SNP distributions consistent with the Hardy–Weinberg equilibrium in both cohorts: UF (p_HWE_ ≥ 0.024) and without UF (p_HW_ ≥ 0.052) (Supplementary Table S3). We applied the Bonferroni correction based on the number of loci studied, p_HW(Bonf)_ ≥ 0.006 [0.05/9]).

Two SHBG-related SNPs demonstrated significant correlations with UF risk: rs3779195 [T/A] *BAIAP2L1* (OR_AA_ = 0.38; 95%CI_AA_ = 0.20–0.91; p_perm(AA)_ = 0.023 [recessive genetic model]; power_AA_ = 81.84%) and rs440837 [A/G] *ZBTB10* (OR_GG_ = 1.93; 95%CI_GG_ = 1.17–3.14; p_perm(GG)_ = 0.010 [recessive genetic model]; power = 85.57%)) (Table 3).

Also, seven SHBG-related SNPs [from the nine studied loci] such as rs3779195 [T/A] *BAIAP2L1*, rs17496332 [A/G] *PRMT6,* rs8023580 [T/C] *NR2F2,* rs780093 [C/T] *GCKR,* rs440837 [A/G] *ZBTB10,* rs10454142 [T/C] *PPP1R21,* and rs7910927 [G/T] *JMJD1C*, interacting amongst themselves (four models of such SNP-SNP_int_ interactions [p_perm_ ≤ 0.01] were found; the cross-validation parameters of these models were as follows: CVC = 10/10, testing balanced accuracy 50.72–56.68%, sensitivity 50.60–65.20%, and specificity 55.70–63.00%), influenced the UF risk (Table 3). Meanwhile, the effects of three loci (rs8023580 [T/C] *NR2F2*, rs780093 [C/T] *GCKR*, and rs10454142 [T/C] *PPP1R21*) were the most serious (75% of the SNP-SNP_int_ models included each of these SNPs) (Table 4). After implementing the Bonferroni correction, the actual p-values for our models substantially exceeded the required thresholds: for three-locus interactions, we observed *p* = 4.88 × 10^−5^; against a threshold of 5.95 × 10^−4^; for four-locus interactions, *p* = 5.86 × 10^−8^; against a threshold of 3.97 × 10^−4^. This considerable margin between the observed values and threshold values confirms the high reliability of these interaction effects.

As a result of the modeling procedure, 16 different UF-significant combinations of SNP-SNP_int_ genotypes were identified (Supplementary Table S4), of which 81.25% (13/16) increased the UF risk, and correspondingly 18.75% (3/16) reduced the risk of UF. Three UF–risk combinations, such as rs8023580-TT *NR2F2*- rs10454142-TC *PPP1R21*- rs780093-TT *GCKR*- rs17496332-AA *PRMT6* (β = 2.027), rs8023580-TT *NR2F2-*rs10454142-TC *PPP1R21*-rs780093-TT *GCKR* (β = 0.924), and rs8023580-TC *NR2F2*-rs7910927-GT *JMJD1C*-rs3779195-TA *BAIAP2L1* (β = 2.669) have the greatest statistical significance (*p* = 0.0007, *p* = 0.006, and *p* = 0.007, respectively) (Supplementary Table S4).

**Figure 1:**
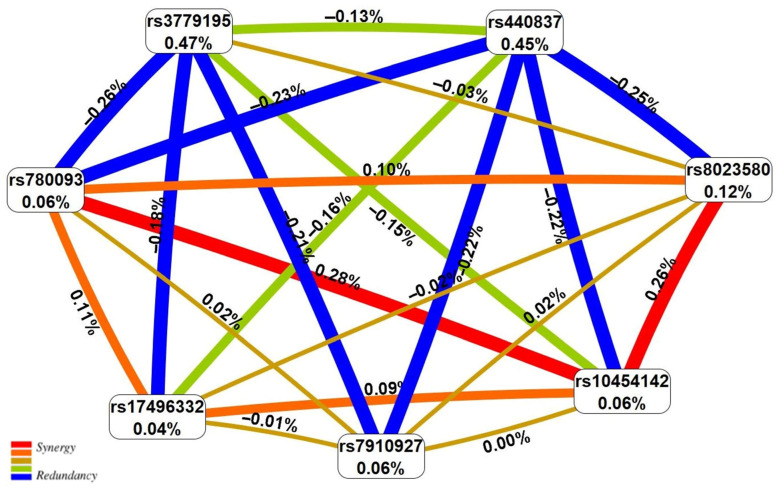
The entropy graph of the UF-associated SNP × SNP interactions (based on the MDR analysis). Positive values of entropy indicate synergistic interactions while the negative values indicate redundancy. The red and orange colors denote strong and moderate synergism, respectively, and the brown color denotes the independent effect; green and blue denote moderate and strong antagonism.

The percentage of UF entropy, depending on the polymorphisms under consideration, was expected to be highest for the two loci rs3779195 [T/A] *BAIAP2L1* (0.47%) and rs440837 [A/G] *ZBTB10* (0.45%), which exhibit the main effect with respect to UF (Figure 1). The effect of the most remarkably paired SNP-SNP_ints_ of both antagonistic (−0.21–−0.26) and synergistic (0.26–0.28%) orientations on the UF risk was almost two times less than the independent exposures of the above two loci (Figure 1).

### 3.1. Potential Functionality of the UF-Associated Polymorphisms

Having identified the associations of SHBG-related polymorphisms with UF, we further assessed the potential functionality of UF-associated loci (and strongly linked SNPs [r^2^ ≥ 0.80]) in the organism (using the in silico approach for this), due to which these polymorphisms may be involved in a susceptibility to UF. We conducted this analysis in two directions: firstly, we examined the functionality of two UF-causal loci (independently associated with UF), rs440837 [A/G] *ZBTB10* (together with 5 proxy variants) and rs3779195 [T/A] *BAIAP2L1* (together with 20 proxy variants); secondly, we evaluated the functionality of all seven UF-associated polymorphisms related with SHBG (both independently and during SNP-SNP_int_) (together with 115 proxy variants).

#### 3.1.1. The Characterization of the Functionality of the Two UF-Causal Loci

SNP rs440837 [A/G] *ZBTB10*

The variant rs440837 [A/G] *ZBTB10* and its five proxy loci are located within functionally significant regulatory regions near *ZBTB10/RP11-48B3.3/RP11-48B3.4* genes that were involved in interaction with 22 transcription factors (TFs). In the liver, both rs440837 [A/G] *ZBTB10* and its proxy locus, rs7013042 *RP11-48B3.4*, were posted in a DNA region where epigenetic modifications of histones (acetylation/methylation) marking potential promoters/enhancers [H3K4me3/H3K4me1] occur (including active promoters/enhancers [H3K9ac/H3K27ac]) (Table 5, Supplementary Table S5).

SNP rs3779195 [T/A] *BAIAP2L1*

rs3779195 [T/A] *BAIAP2L1* and its 20 LD SNPs were extremely significant for the DNA (in the *BAIAP2L1/BRI3* gene region) collaboration regulation with 68 TFs (17 SNPs) and 18 regulatory proteins (4 SNPs) (Table 5, Supplementary Table S5), and affect the transcription of 15 genes (Table 4, Supplementary Table S6, Supplementary Table S7) and the splicing of 3 genes (*BRI3*, *TECPR1*, *BAIAP2L1*) (Table 5, Supplementary Table S8, Supplementary Table S9) including both organs of the female reproductive system (uterus [eQTL-*RP11-307C18.1*], ovary [eQTL-*RP11-307C18.1*]) and other organs directly related to the UF pathophysiology: adipose tissue [eQTL-*BRI3*, *RP11-307C18.1*; sQTL-*BRI3*], musculoskeletal tissue [eQTL-*BRI3*, *RP11-307C18.1*, *ASNS*, *BAIAP2L1*; sQTL-*BRI3*], the brain [eQTL-*BHLHA15*, *RP11-307C18.1*; sQTL-*TECPR1*, *BRI3*], adrenal glands [eQTL-*RP11-307C18.1*], the thyroid gland [eQTL-*BAIAP2L1*, *RP11-307C18.1*, *TECPR1, BHLHA15, LMTK2*; sQTL-*BRI3*], the blood [eQTL-*TECPR1*, *RP11-307C18.1*], etc. In the liver, both rs3779195 [T/A] *BAIAP2L1* and several of its proxy SNPs were located in genomic regions where various histones’ epigenetic modifications (acetylation/methylation) were present, such as H3K4me3 (labeling the promoters [3 SNPs]), H3K4me1 (marking the enhancers [10 SNPs]), H3K9ac (labeling the active promoters [6 SNPs]), and H3K27ac (marking the active enhancers [8 SNPs]) (Table 5, Supplementary Table S5). Also, in the liver, rs3779195 [T/A] *BAIAP2L1* and 17 proxy SNPs were linked with the expression of two genes (*BRI3* and *RP11-307C18.1*). Interestingly, UF-protective allele A rs3779195 [T/A] *BAIAP2L1* was associated with high *RP11-307C18.1* transcription (NES:−0.54) and low *BRI3* expression (NES:0.87) in the liver (Supplementary Table S6).

After characterizing the functionality of the two UF-causal loci and their proxy SNPs, we then evaluated the protein–protein communications (PPcs) linked with these loci in the STRING program. For example, the PPc of 22 TFs and three proteins encoded by the genes *ZBTB10*, *RP11-48B3.3*, and *RP11-48B3.4* (a total of 25 proteins) were studied for the locus rs440837 [A/G] *ZBTB10*, while the PPc of 68 TFs, 18 regulatory proteins, and 15 proteins encoded by the genes *LMTK2*, *TECPR1*, *AC004967.7*, *ASNS*, *BAIAP2L1*, *BRI3*, *RP11-307C18.1*, *RP11-307C18.2*, *RP11-307C18.3*, *RP11-307C18.4*, *RP11-307C18.5*, *RP11-307C18.6*, *RP11-307C18.7*, *RP11-307C18.10*, and *RP11-307C18.11* (a total of 101 proteins) were considered for the locus rs3779195 [T/A] *BAIAP2L1*. Additionally, several proteins most significant for these PPcs (according to the STRING data) were included in the PPc analysis of these two UF-causal loci.

**Figure 2:**
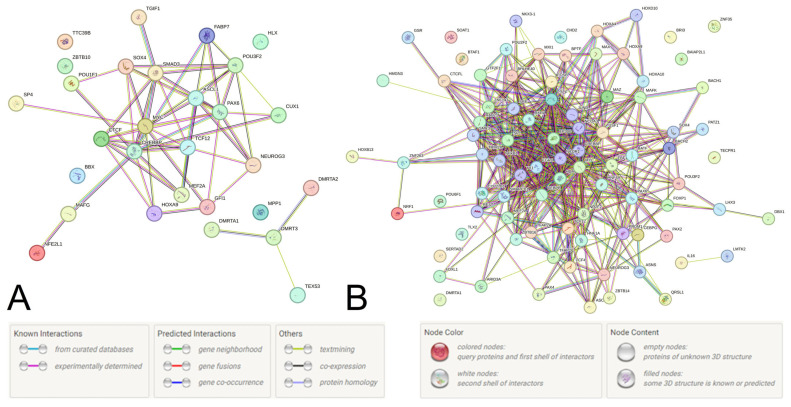
Protein–protein communications determined by the UF-causal SNPs rs440837 [A/G] *ZBTB10* (**A**) and rs3779195 [T/A] *BAIAP2L1*5 (**B**) and their proxy loci (STRING program data).

Among the many identified pathways involving PPc linked with two UF-causal loci (over 350 pathways for rs440837 [A/G] *ZBTB10* (Figure 2A, Supplementary Table S10) and over 350 pathways for rs3779195 [T/A] *BAIAP2L1* (Figure 2B, Supplementary Table S11)), various regulatory influences on gene transcription (including estrogen-dependent gene expression and miRNA transcription regulation, etc.), steroid hormone processes (levels, biosynthesis, androgen receptor signaling, etc.), morphogenesis (including gonad development, apoptotic process, etc.) prevail and are characterized by the TGF-beta signaling pathway (hsa04350). All of the above pathways may be important for the pathophysiology of UF.

#### 3.1.2. The Characterization of the Seven UF-Associated Loci Functionality

The overwhelming majority of UF-related loci (118 out of 122 considered variants [96.72%] such as seven UF-associated polymorphisms and 115 strongly linked SNPs) had pronounced potential epigenetic influences on 13 adjoining genes (*GCKR*, *KLRAQ1*, *FOXN2*, *NR2F2*, *BRI3*, *RP11-327J17.2*, *BAIAP2L1*, *PPP1R21*, *PRMT6*, *RP11-327J17.3*, *ZBTB10*, *JMJD1C,* and *RP11-48B3.4*) due to their localization in such regulatorily valuable regions of these genes as (a) open chromatin (they are highly sensitive to the effects of DNase enzyme) (21.31%/26 variants); (b) evolutionarily conservative regions (4.10%/5 variants); (c) promoters (8.19%/10 variants); (d) enhancers (22.95%/28 variants); (e) regulatory motifs (90.16%/110 variants); and (f) liaison sites with regulatory proteins (13.93%/17 variants) (Supplementary Table S5).

More than 95% of UF-correlated loci (116 out of 122 variants [95.08%]) were communications to the transcription activity of 47 genes (Supplementary Tables S6 and S7). The abovementioned loci were eQTL impact promoters in several organs including both organs of the female reproductive system (uterus [*RP11-460M2.1*, *RP11-307C18.1*] and ovary [*RP11-307C18.1*]) and other UF-significant organism organs, such as adipose tissue [*NRBP1*, *ATRAID*, *REEP3*, *BRI3*, *GTF2A1L*, *KRTCAP3*, *PPM1G*, *STON1-GTF2A1L*, *PPP1R21*, *MRPL35P2*, *PRMT6*, *RP11-307C18.1*], the brain (pituitary) [*RP11-307C18.1*, *FOXN2*, *GTF2A1L*, *MRPL35P2*], thyroid [*KRTCAP3*, *AC074117.10*, *IFT172*, *ATRAID*, *GTF2A1L*, *BAIAP2L1*, *MRPL35P2*, *C2orf16*, *PRMT6*, *FNDC4*, *GCKR*, *JMJD1C-AS1*, *LMTK2*, *PPM1G*, *ZNF512*, *STON1*, *PPP1R21*, *REEP3*, *RPL7AP50*, *RP11-307C18.1*, *TECPR1*], the blood [*RP11-307C18.1*, *KRTCAP3*, *PRMT6*, *TECPR1*, *NRBP1*], adrenal glands [*FOXN2*, *GTF2A1L*, *KRTCAP3*, *MRPL35P2*, *PRMT6*, *RP11-307C18.1*], and also in the liver where SHBG is formed [*RP11-307C18.1*, *RP11-307C18.2*, *PRMT6*, *GTF2A1L*, *RP11-327J17.2*, *BRI3*] (Supplementary Tables S6 and S7).

A substantial proportion of the UF-related loci (34 out of 122 SNPs [27.87%]) showed significant associations with the splicing control of 13 genes (*GPN1*, *KRTCAP3*, *PPP1R21*, *IFT172*, *BRI3*, *STON1*, *FNDC4*, *TRIM54*, *STON1-GTF2A1L*, *GTF2A1L*, *BAIAP2L1*, *GCKR,* and *SNX17*), and they showed their sQTL influences in female reproductive system organs (the uterus [*IFT17*, *PPP1R21*], the ovaries [*IFT17*, *FNDC4*]) and other such UF-important organs as the brain (pituitary) [*IFT172*, *FNDC4*, *PPP1R21*], adipose tissue [*GPN1*, *BRI3*, *SNX17*, *FNDC4*, *IFT172*, *GTF2A1L*, *STON1*, *PPP1R21*, *STON1-GTF2A1L*], adrenal glands [*GCKR*, *FNDC4*, *IFT172*], the thyroid gland [*IFT172*, *BRI3*, *PPP1R21*, *KRTCAP3*], and also the liver [*GCKR, FNDC4*] (Supplementary Tables S8 and S9).

**Figure 3:**
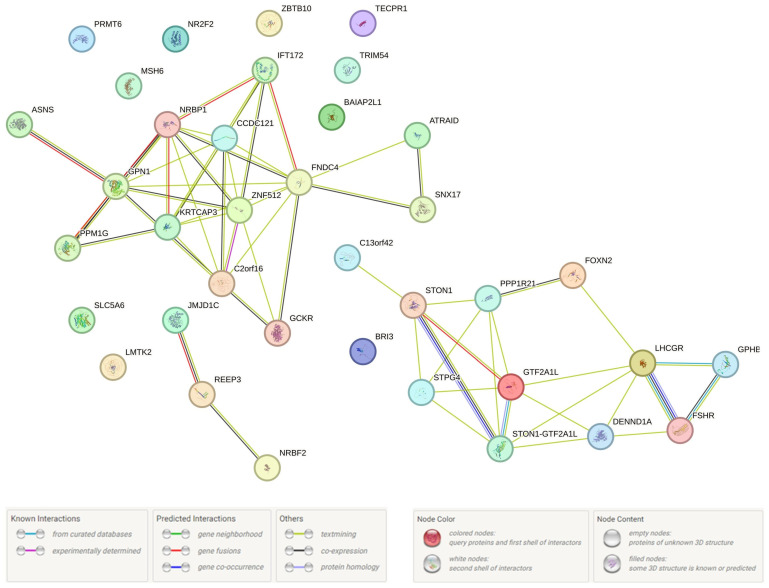
Interaction of UF-correlated proteins (STRING program data).

In the final stage of our study, we investigated PPcs involving the protein products of 52 UF candidate genes (functionally correlated with seven UF-causal loci and 115 LD SNPs, the data are presented above). The data obtained (Figure 3) indicate the hub value of PPc of hormone-related genes (such as *LHCGR*, *FSHR*, *STON1,* and *STON1-GTF2A1L*), and the involvement of PPc in the regulation of gene transcription (due to transcription factor IIA (alpha/beta subunits) [p_FRD_ = 0.0280, SM01371]), as well as interactions with hormone ligand-binding receptors [p_FRD_ = 0.0062,HSA-375281] and the content control of SHBG [p_FRD_ = 3.11 × 10^−6^, EFO: 0004696], testosterone [p_FRD_ = 2.83 × 10^−6^, EFO: 0004908], liver enzymes [p_FRD_ = 0.0001, EFO: 0004582], fasting blood glucose [p_FRD_ = 0.0038, EFO:0004465] [p_FRD_ = 2.60 × 10^−8^, EFO: 0011008], lipids [p_FRD_ = 2.13 × 10^−5^,EFO: 0004529], etc. All of the aforementioned pathways may impact the biology of UF.

## 4. Discussion

In this study, we have proven for the first time the risk (OR = 1.93)/protective effect (OR = 0.38) for UF of SHBG_con_-increasing (allele G rs440837 [A/G] *ZBTB10*)/decreasing (allele A rs3779195 [T/A] *BAIAP2L1*) genetic variants of GWAS-significant for SHBG_con_ polymorphisms. The susceptibility to UF was also determined by the interactions of seven SHBG-related SNPs, which, while showing potential pronounced functionality, were involved in UF impact pathways of gene transcription regulation, interaction with hormone ligand-binding receptors, the content control of SHBG, testosterone, liver enzymes, fasting blood glucose, lipids, etc.

Our findings demonstrate that the AA genotype of rs3779195 [T/A] *BAIAP2L1* has a protective effect against UF development (if it is present in the genotype, the risk of UF decreases by more than 60% [OR = 0.38]). It should be noted that our data on associations of rs3779195 [T/A] *BAIAP2L1* with UF were obtained for the AA genotype, which is quite rare and occurs in patients (1.93%) two times less frequently than in the control (3.80%), which led to a pronounced OR index equal to 0.38. The results of two previously published GWASs showed a correlation of the allele A with low SHBG_con_ [18,21]. So, the SHBG-lowering genetic variant (allele A rs3779195 [T/A] *BAIAP2L1*) (data from the two abovementioned GWAS) was UF-protective (our results). The rs3779195 [T/A] *BAIAP2L1* polymorphism and its proxy loci may have pronounced functionality—they were involved in plenty of (n = 86) “DNA-TFs/protein-regulatory” cooperations (in the *BAIAP2L1/BRI3* genes region), in the regulation of the eQTL (15 genes) and sQTL (3 genes) traits in various organs of both the female reproductive system (uterus [eQTL-*RP11-307C18.1*], ovary [eQTL-*RP11-307C18.1*]), and other organs directly related to UF pathophysiology (adipose; skeletal muscles; blood; adrenal/thyroid glands;brain; and others), including in the liver (*BRI3; RP11-307C18.1* [eQTL]) (our in silico data), which is the major organ of SHBG formation in the body [54].

The protein product of the *BAIAP2L1* gene (brain-specific angiogenesis inhibitor 1—associated protein 2-like 1 [BAIAP2L1], also known as insulin receptor tyrosine kinase substrate [IRTKS]), is involved in a number of UF-significant processes such as morphogenesis and cell migration (involved in the protrusion of the plasma membrane and the formation of actin), proliferation, and apoptosis (due to the activation of EGFR-ERK, PI3K/AKT, and other pathways) [55,56,57]. These processes play an important role in tumorigenesis [56,57], including in malignant neoplasms of the female reproductive system (ovarian cancer) [55]. Interestingly, positive genetic correlations are observed between UF and ovarian cancer [58], and it can be assumed that one of the genes that may underlie these correlations may be the *BAIAP2L1* gene. Importantly, several rs3779195 [T/A] *BAIAP2L1* LD variants have also been correlated (GWAS materials) with SHBG_con_ and testosterone level: SHBG_con_—rs112758337 (D’ = 1.00; r^2^ = 0.96), rs1688606 (D’ = 1.00; r^2^ = 0.96), and rs4268041 (D’ = 1.00; r^2^ = 0.99) [21]); total testosterone—rs1635612 (D’ = 1.00; r^2^ = 0.96) [21] and rs35903783 (D’ = 1.00; r^2^ = 0.41) [20]. So, the genome region in the SNP rs3779195 [T/A] *BAIAP2L1*, due to its assumed pronounced functional meaning in silico (epigenetic/eQTL effects) in the liver (the main organ of SHBG synthesis), is essential in SHBG_con_/testosterone level regulation in the organism and, thanks to this, may affect UF risk.

The present study revealed the risk effect of the genotype GG rs440837 [A/G] *ZBTB10* on UF development (its presence in the genotype increased UF risk by almost two times [OR = 1.93]). It should be noted that our data on associations of rs440837 [A/G] *ZBTB10* with UF were received for the GG genotype, which occurs with a low frequency and its prevalence in patients (7.03%) is 1.6 times higher than in the control (4.31%), which led to an expressed OR index of 1.93. The allele variant G rs440837 [A/G] *ZBTB10* showed associations with high SHBG_con_ in two previously presented GWASs [18,21]. So, the SHBG_con_-boosting genetic variant (allele G rs440837 [A/G] *ZBTB10,* in the result of the two abovementioned GWASs) was UF-risky (the results of this study). The UF-causal locus rs440837 [A/G] *ZBTB10* (jointly with LD SNPs) was potentially impactful for the “DNA-TFs engagement” (n = 22 TFs) and other epigenetic modifications (histone acetylation/methylation) at the *ZBTB10/RP11-48B3.3/RP11-48B3.4* genes’ place in liver (our in silico results). It is important that the protein of the same name, encoded by the *ZBTB10* gene, is an important regulator of gene transcriptional activity due to its modulating effects on the binding of RNA polymerase II to the corresponding genomic sequence [59], which may be important for the regulation of SHBG synthesis in the liver. Interestingly, a number of loci strongly associated with rs440837 [A/G] *ZBTB10* also showed their GWAS relevance for SHBG_con_, with such loci as rs72688090 (D’ = 0.85; r^2^ = 0.33) [60], rs388922 (D’ = 0.96; r^2^ = 0.53), rs575452 (D’ = 0.71; r^2^ = 0.28), and rs117921873(D’ = 1.00; r^2^ = 0.26) [21]. So, we would like to note the importance of the rs440837 [A/G] *ZBTB10* genome region in regulating SHBG_con_ (due to the supposedly significant in silico epigenetic influences in the liver, in which SHBG is mainly formed) and its involvement in UF biology/risk due to this.

Summarizing the material obtained in the work (presented above), it can be concluded that SHBG_con_-lowering genetic variant (AA genotype rs3779195 [T/A] *BAIAP2L1*) reduces UF risk (OR = 0.38), and SHBG_con_-raising genetic variant (genotype GG rs440837 [A/G] *ZBTB10*) increases UF risk (OR = 1.93). There is only one paper in the literature on this topic with extremely contradictory results: after conducting an MR, the authors did not identify a link between SHBG and UF in the GWAS meta-analysis of the data from two cohorts, FinnGen and FibroGENE, and found an inverse correlation between them in one of these cohorts—FibroGENE [23]. At the same time, in the work of Misao et al., based on experimental data, a higher level of SHBG in UF cells compared to normal myometrium was found in the vast majority of the biological samples studied (21 out of 23, 91%) [61], which is completely consistent with our results and differs from the abovementioned data obtained by Wang et al. in their MR analysis [23].

It should be noted that the possible reasons for the differences between our results and the data of Wang et al. obtained by MR of UF [23], may include the following: firstly, in the work of Wang et al., an MR analysis was performed based on the combined GWAS data for UF (FinnGen and FibroGENE) and SHBG (UK Biobank [20]); the pleiotropic effects of the individual most SHBG-significant loci in both UF and UF-significant risk factors (for example, the age of menarche/menopause, etc.), through which the UF-significant effects of SHBG-significant loci may be mediated, were not considered; whereas, in our work, the independent effects of the two SHBG-significant loci in UF were found. As an example of the possibility of such “discrepancies” in the results of even one study, one can cite the work of Garitazelaia et al. [62], in which the MR analysis did not reveal reliable causal relationships between the content of sex hormones and endometriosis, but at the same time (and this was a surprise to the authors!) pleiotropic genetic associations of two loci of the *FSHB* gene (rs11031002, rs11031005) with endometriosis and sex hormone concentrations were found; the SNPs of the *FSHB* gene region (rs11031005, rs11031006) also demonstrated significant pleiotropic associations mediating endometriosis and related signs (menarche/menopause age and menstrual cycle duration) [62]. Secondly, our results may depend to a certain extent on a number of comorbid conditions common in the sample we studied. For example, more than 1/3 of the patients studied in our work (36.38%) have endometriosis (Table 1). At the same time, endometriosis is characterized by a clear association between high SHBG and an increased risk of disease [63,64]. Using the MR analysis by Qu et al. (the study analyzed GWAS data from the FinnGen cohort) showed that endometriosis is associated with an increased risk of UF, while PCOS (unlike endometriosis, low SHBG is risky for PCOS) was associated with a reduced risk of UF [65]. Thirdly, in the work of Wang et al., an inverse correlation between SHBG and UF was observed only in FibroGENE (in the MR analysis of this cohort, BMI was adjusted due to the similarity of GWAS data on SHBG and UF), and not in FinnGen, and as the authors themselves note, “care needs to be taken for bias resulting from sample overlap and collider bias caused by adjusting BMI” [23]. A critical difference that may underlie these inconsistent findings is the unequal number of instrumental variables used across the two cohorts. In the FinnGen analysis, fewer SHBG-associated SNPs were available or retained after quality control and filtering for linkage disequilibrium and pleiotropy. This reduced number of instruments likely weakened the statistical power and increased vulnerability to weak instrument bias, contributing to the null result. In contrast, the FibroGENE analysis utilized a larger set of instruments, enabling more stable estimates. However, this analysis also involved sample overlap with the SHBG GWAS, raising the possibility of bias from overfitting or collider effects, especially in the context of multivariable MR adjusting for genetically correlated traits such as BMI. These discrepancies in instrument number and dataset composition likely contributed to the divergent outcomes observed across the two cohorts. In addition, it is important to note that the information identified in the work of Wang et al., the nominally significant causal relationship between a higher level of SHBG and a lower UF risk, according to the authors themselves (!) contradicts one of the main conclusions made in their work—“ the protective effect of a higher level of total testosterone on uterine leiomyoma, as higher SHBG usually means less bioactive testosterone” [23]. Interestingly, Wang et al. also reported an inverse association between total testosterone and fibroid risk, a conclusion that aligns with our findings. However, their interpretation of the nominally inverse relationship between SHBG and fibroids as evidence for a protective effect of SHBG contradicts the well-established inverse relationship between SHBG and free testosterone. This inconsistency, combined with the methodological limitations discussed above, underscores the need for the cautious interpretation of MR findings, particularly in hormonally driven conditions with complex genetic architectures and interrelated exposures (it should be emphasized that this conclusion is made by Wang et al. and the protective role of testosterone in UF is fully consistent with the results/assumptions of our work, detailed below). Thus, there is an extremely pronounced ambiguity in the currently limited genetic data on the relationship between the genetic determinants of SHBG and UF (even in the framework of one study, for example, the Wang et al. MR analysis [23]), and further research in this area is needed.

One potential mechanism underlying the association of SHBG with UF involves the transportation/deposition of testosterone [12]. Current evidence estimates that approximately 80% of testosterone is in the SHBG-bound state, and only 1% is a free fraction and exhibits biological activity [12]. Thus, SHBG_con_, by directly affecting the level of bound/free testosterone, will largely determine its effects on UF. A significant association of “SHBG–testosterone” is also evidenced by the indicators of a pronounced negative genetic correlation of SHBG with the level of free testosterone, which in women reaches a value of −0.75 [20,66]. In this case, the effects of testosterone can be realized by both its free (bioactive) and bound fractions [14]. It is assumed that testosterone bound to SHBG can enter cells by endocytosis and be released as a result of pH changes [14].

The existing literature presents limited and conflicting data regarding the association of testosterone with UF risk. For example, in the work of Wong et al., a high content of bioavailable testosterone was demonstrated to be associated with both an increased UF risk (OR = 1.33) and, concurrently, a low UF recurrence risk [13]. On the one hand, a number of studies indicate that testosterone, transformed by aromatase into estradiol in myomatous cells, promotes the development of local hyperestrogenism in the UF area, which stimulates the growth of myomatous nodes [12]. On the other hand, there is evidence of testosterone’s protective value in UF [23]. Wang et al., working with MR GWAS data from two cohorts, FinnGen and FibroGENE, revealed a reverse relationship between total testosterone and UF in each of these cohorts, as well as in their combined analysis (OR = 0.90); no correlations between free testosterone and UF were found [23]. These data disagree with the currently available unambiguous ideas about a significantly positive relationship between total and free testosterone levels: according to Ruth et al., in women the genetic correlations between these parameters are 0.65 [20]. As one of the possible reasons for the lack of a genetic link between free testosterone and UF (Wang et al.), it is suggested that in the GWAS materials Ruth et al. used in their study, data on bioavailable testosterone levels were not obtained by direct measurements, but were calculated using the Vermeulen formula (based on data on testosterone, SHBG, and albumin), and these estimates, based on model formulas regarding the binding ability of SHBG, may not correspond to the true affinity of SHBG for binding to testosterone [23].

A review article by Whitton and Baber notes the proapoptotic/antiproliferative testosterone effects on hormone-dependent organs/structures of the female reproductive system, such as the mammary gland and uterus endometrium [66], despite the fact that their cells (like myomatous cells) contain high levels of both aromatases and androgen receptors [23,67]. The proapoptotic/antiproliferative effects of testosterone may be based on various testosterone-related biological mechanisms such as the activation of the growth and maturation of primordial follicles, increased metabolic processes in oocytes (the early stages of folliculogenesis), the suppression of follicle growth, the inhibition of estrogen formation (the late stages of folliculogenesis), the stimulation of corpus luteum formation, the increased synthesis of progesterone (luteal phase) etc. [68,69].

Notably, the observed association between low testosterone concentrations (both total and free; the genetic correlations between them in women are 0.65 [20]) and increased UF risk may be mediated through established UF risk factors. These include obesity as evidenced in our cohort by positive BMI-UF association (Table 1) and cardiovascular diseases, etc. [1,2,3]. Thus, low levels of total testosterone correlate with an increased risk of metabolic syndrome, obesity/dyslipidemia, hypertension, and insulin resistance/diabetes [14].

Another potential mechanism explaining the association of SHBG-lowering/SHBG-increasing genetic variants with low/high UF risk may be the “genetic” association of SHBG with estradiol [20]. Thus, according to a large-scale study by Ruth et al., there are significant positive genetic correlations between SHBG and estradiol levels in women (0.45) [20]. Estradiol is a known driver of the growth of myomatous nodules [13]. Based on these data, it can be assumed that a low level of SHBG will be genetically correlated with a low content of estradiol [20], which will have a protective value for UF [13], and, conversely, a high level of SHBG, genetically correlated with a high concentration of estradiol, will contribute to an increase in UF risk, which is fully consistent with the results we have obtained. Also, in the work of Ruth et al., negative genetic correlations have been shown between the level of estradiol and the content of both total (−0.25) and free (−0.51) testosterone [20], which is also fully consistent with the assumption we made above (according to the results obtained in this work) about the SHBG-induced protective effect of testosterone on UF risk (which is opposite to the effect of estradiol).

So, the biomedical justification for the established UF-protective role of the SHBG-reducing genetic variant rs3779195 [T/A] *BAIAP2L1* (genotype AA,OR = 0.38) may be the pronounced proapoptotic/antiproliferative effects of elevated free testosterone levels recorded at low SHBG_con_, and, conversely, the value of the UF risk of the SHBG-enhancing genetic variant rs440837 [A/G] *ZBTB10* (genotype GG,OR = 1.93), identified in our work, may be associated with the “weak” proapoptotic/antiproliferative effects of low levels of free testosterone, typical for a high SHBG_con_.

Notably, this genetic panel (nine loci linked to SHBG levels) has been previously investigated in our studies of breast cancer [70,71,72] and endometriosis [73,74]. According to the findings from our previous work, the risk of developing breast cancer was influenced by rs10454142 *PPP1R21* [71], while the likelihood of developing endometriosis was determined by rs440837 [A/G] *ZBTB10* [73]. Based on these data, we can see that rs440837 [A/G] *ZBTB10* was associated with both endometriosis (as reported by Ponomareva et al. [73]) and UF (according to this study). It may be a syntropic SNP/gene that influences the development of uterus-benign proliferative conditions, such as UF and endometriosis. This SNP/gene may play a significant role in determining the “common” genetic susceptibility to these conditions, as suggested by previous studies [75,76,77].

This work has a number of limitations, which include the following: (a) we obtained the results of the association of SHBG_con_ to significant genetic variants with UF and data on the pronounced effects of individual loci (OR = 0.38 for genotype AA rs3779195 [T/A] *BAIAP2L1* and OR = 1.93 for genotype GG rs440837 [A/G] *ZBTB10*) in only one sample, and their replication/verification in another independent cohort is necessary; (b) UF-significant associations of polymorphisms linked with SHBG_con_ were obtained only in one mono-ethnic cohort of Europeans (Russians from central Russia) and confirmation of these associations in populations of other ethnic groups is needed due to the fact that the potential stratification of the population by ethnic composition can have a significant impact on the results of associative genetic studies; (c) the possible functionality of UF-significant polymorphisms (and strongly linked loci) was predicted by us only in silico, and experimental studies are needed to confirm these assumptions in silico; (d) the causal relationships between the genetic determinants of SHBG_con_ and UF, which we assumed in this work, need additional confirmation by Mendelian randomization; (e) there is a certain probability of non-detection of UF (for example, small-sized UF, cervical UF, etc.) during ultrasound examination of women in the control group.

It should be noted that with the existing paucity and ambiguity/inconsistency of data on the topic of “SHBG-UF” and “testosterone-UF” correlations, there is an obvious need to continue research in this area in order to accumulate experimental data on the issue of genetic links between SHBG, testosterone, and UF, their further generalization, and the establishment of unambiguous patterns existing between them. Therefore, our work is actually the first experimental genetic study on this topic.

## 5. Conclusions

SHBG_con_-significant genetic determinants were associated with UF risk.

## Figures and Tables

**Table 1:** The characteristics of participants from the case and control groups.

Parameters	Cases (n = 569) $\bar{X}$ ± SD/% (n)	Controls (n = 973) $\bar{X}$ ± SD/% (n)	*p*
Age, years	43.22 ± 8.35	40.26 ± 8.53	<0.001
Height, m	1.66 ± 0.06	1.66 ± 0.06	>0.05
Weight, kg	76.43 ± 14.35	70.54 ± 13.25	<0.001
BMI, kg/m^2^	27.90 ± 5.38	25.22 ± 4.52	<0.001
Proportion of the participants by relative BMI, % (n):			
underweight (<18.50)	1.58 (9)	3.60 (35)	<0.001
normal weight (18.50–24.99)	31.81 (181)	54.98 (535)	
overweight (25.00–29.99)	34.27 (195)	27.85 (271)	
obese (>30.00)	32.34 (184)	13.57 (132)	
Family history of UF (mother had UF)	35.15 (200)	17.06 (166)	<0.001
Married	85.06 (484)	85.92 (836)	>0.05
Smoking (yes)	13.71 (78)	17.06 (166)	>0.05
Drinking alcohol (≥7 drinks per week)	2.99 (17)	3.08 (30)	>0.05
Oral contraceptive use	9.49 (54)	10.07 (98)	>0.05
Age at first oral contraceptive use (mean, years)	23.43 ± 2.28	23.61 ± 2.34	>0.05
Age at menarche and menstrual cycle			
Age at menarche, years	13.45 ± 1.31	13.29 ± 1.26	>0.05
Proportion of the participants by relative age at menarche, % (n) early (<12 years) average (12–14 years) late (>14 years)	4.62 (26) 80.28 (452) 15.10 (85)	6.17 (60) 80.06 (779) 13.77 (134)	>0.05
Duration of bleeding menstrual (mean, days)	5.15 ± 1.56	4.96 ± 0.95	>0.05
Menstrual cycle length (mean, days)	28.04 ± 2.15	28.18 ± 2.25	>0.05
Reproductive characteristic			
Age at first birth (mean, years)	21.19 ± 2.59	21.69 ± 3.48	>0.05
Time since last birth (mean, years)	15.08 ± 2.28	14.31 ± 2.07	>0.05
Gravidity (mean)	3.34 ± 2.22	2.42 ± 1.53	<0.001
No. of births (mean)	1.46 ± 0.85	1.50 ± 0.66	>0.05
No. of spontaneous abortions (mean)	0.26 ± 0.64	0.23 ± 0.50	>0.05
No. of induced abortions (mean)	1.59 ± 1.65	0.66 ± 0.97	<0.001
No. of induced abortions: 0 1 2 3 ≥4	31.81 (181) 23.20 (132) 21.62 (123) 12.30 (70) 11.07 (63)	58.99 (574) 23.74 (231) 10.18 (99) 5.45 (53) 1.64 (16)	<0.001
History of infertility	13.71 (78)	5.14 (50)	<0.001
Gynecological pathologies			
Cervical disorders	26.01 (148)	25.18 (245)	>0.05
History of sexually transmitted diseases	27.06 (154)	26.93 (262)	>0.05
Chronic endometritis	10.02 (57)	5.65 (55)	<0.01
Chronic inflammation of adnexa	34.62 (197)	31.96 (311)	>0.05
Endometrial hyperplasia	47.10 (268)	-	-
Endometriosis	36.38 (207)	-	-
Adenomyosis	21.27 (121)	-	-

BMI, body mass index.

**Table 2:** The GWAS data on associations of the studied polymorphisms of the candidate genes with the SHBG_con_ and testosterone concentrations.

SNP, Gene (Chromosome Position (hg38))	Phenotype	Association (Significance) (Affected Allele)	Reference
rs17496332 *PRMT6* (1p13.3)	SHBG	β = −0.028 (*p* = 1 × 10^−11^) (A)	[18]
rs780093 *GCKR* (2p23.3)	SHBG	β = −0.032 (*p* = 2 × 10^−16^) (T)	[18]
rs10454142 *FOXN2* (2p16.3)	SHBG	β = 0.026 (*p* = 1 × 10^−7^) (T)	[18]
rs3779195 *BAIAP2L1* (7q19.3)	SHBG	β = −0.033 (*p* = 3 × 10^−8^) (A)	[18]
		β = −2.41 (*p* = 9 × 10^−9^) (A)	[21]
rs440837 *ZBTB10* (8q19.13)	SHBG	β = −0.030 (*p* = 3 × 10^−9^) (A)	[18]
		β = 1.43 (*p* = 1 × 10^−12^) (G)	[21]
rs7910927 *JMJD1C* (10q19.3)	SHBG	β = −0.048 (*p* = 6 × 10^−35^) (T)	[18]
rs4149056 *SLCO1B1* (12p12.1)	SHBG	β = 0.029 (*p* = 2 × 10^−8^) (T) β = 0.030 (*p* = 1 × 10^−73^) (T) β = −1.23 (*p* = 7 × 10^−29^) (C) β = −0.065 (*p* =5 × 10^−48^) (C)	[18] [20] [21] [22]
	total testosterone	β = 0.028 (*p* = 5 × 10^−10^) (C) β = −0.029 (*p* = 1 × 10^−14^) (T)	[22] [20]
	bioavailable testosterone	β = 0.02 (*p* = 2 × 10^−16^) (C) β = −0.043 (*p* = 3 × 10^−35^) (T)	[21] [20]
rs8023580 *PPP1R19* (15q26.2)	SHBG	β = −0.03 (*p* = 8 × 10^−12^) (T)	[18]
rs11950660 *SHBG* (17p13.1)	SHBG	β = 0.103 (*p* = 2 × 10^−106^) (T)	[18]
		β = 6.14 (*p* = 1 × 10^−300^) (T)	[21]
		β = 3.9 (*p* = 2 × 10^−75^) (T)	[16]
	total testosterone	β = 31.8 (*p* = 1 × 10^−41^) (T)	[16]

**Table 3:** Associations of the SHBG-significant gene polymorphisms with UF.

SNP	Gene	Minor Allele	n	Allelic Model				Additive Model				Dominant Model				Recessive Model			
				OR	95%CI		*p*	OR	95%CI		*p*	OR	95%CI		*p*	OR	95%CI		*p*
					L95	U95			L95	U95			L95	U95			L95	U95	
rs17496332	*PRMT6*	G	1452	0.94	0.80	1.10	0.410	0.93	0.78	1.11	0.429	0.90	0.70	1.15	0.387	0.94	0.66	1.34	0.729
rs780093	*GCKR*	T	1470	1.08	0.93	1.26	0.304	1.10	0.93	1.31	0.279	1.13	0.88	1.46	0.339	1.14	0.83	1.57	0.427
rs10454142	*PPP1R21*	C	1445	0.96	0.81	1.13	0.607	1.01	0.84	1.22	0.929	0.98	0.77	1.25	0.873	1.10	0.73	1.66	0.639
rs3779195	*BAIAP2L1*	A	1452	1.09	0.90	1.33	0.372	1.14	0.91	1.43	0.244	1.29	1.00	1.67	0.054	**0.38**	**0.20**	**0.91**	**0.023**
rs440837	*ZBTB10*	G	1423	1.06	0.88	1.26	0.539	1.11	0.91	1.36	0.292	1.01	0.78	1.29	0.963	**1.93**	**1.17**	**3.14**	**0.010**
rs7910927	*JMJD1C*	T	1471	0.92	0.79	1.07	0.258	0.88	0.75	1.05	0.161	0.81	0.62	1.06	0.131	0.89	0.67	1.19	0.446
rs4149056	*SLCO1B1*	C	1418	0.99	0.83	1.19	0.943	0.98	0.80	1.21	0.870	0.99	0.77	1.28	0.961	0.91	0.52	1.60	0.739
rs8023580	*NR2F2*	C	1451	0.99	0.84	1.17	0.906	1.00	0.83	1.21	0.995	1.05	0.82	1.33	0.719	0.87	0.56	1.35	0.529
rs12150660	*SHBG*	T	1486	1.00	0.84	1.19	0.982	0.94	0.77	1.14	0.522	0.95	0.75	1.21	0.686	0.82	0.50	1.34	0.430

All results were obtained after adjustment for covariates. *p* values < 0.05 are shown in bold. Abbreviations: OR, odds ratio; 95%CI, 95% confidence interval.

**Table 4:** SNP × SNP interactions of SHBG-significant genes associated with UF.

N	SNP × SNP Interaction Models	MB-MDR Data							GMDR Data (Model Cross-Validation)				
		NH	betaH	WH	NL	betaL	WL	p_perm_	OR (95%CI)	TBA	S_e_	S_p_	CVC
Three-order interaction models (*p* = 4.88 × 10^−5^)													
1	rs8023580 *NR2F2*-rs7910927 *JMJD1C*-rs3779195 *BAIAP2L1*	3	0.740	18.74	-	-	-	0.001	1.75 (1.37–2.23)	50.72	50.60	60.84	10/10
2	rs8023580 *NR2F2*-rs10454142 *PPP1R21-*rs780093 *GCKR*	4	0.530	16.49	3	−0.549	10.45	0.01	1.76 (1.43–2.18)	54.51	53.08	60.95	10/10
Four-order interaction models (*p* = 5.86 × 10^−8^)													
3	rs440837 *ZBTB10*-rs10454142 *PPP1R21*-rs780093 *GCKR*-rs17496332 *PRMT6*	7	1.168	30.61	2	−0.841	9.89	0.001	2.06 (1.67–2.55)	53.44	54.83	63.00	10/10
4	rs8023580 *NR2F2*-rs10454142 *PPP1R21*-rs780093 *GCKR*-rs17496332 *PRMT6*	5	1.380	29.41	1	−0.974	3.06	0.001	2.35 (1.90–2.92)	56.68	65.20	55.70	10/10

The results were obtained using the MB-MDR method with adjustment for covariates. NH, the number of significant high-risk genotypes in the interaction; betaH, the regression coefficient for high-risk exposition in the step2 analysis; WH, the Wald statistic for the high-risk category; NL, the number of significant low-risk genotypes in the interaction; betaL, the regression coefficient for low-risk exposition in the step2 analysis; WL, the Wald statistic for the low-risk category; p_perm_, the permutation *p*-value for the interaction model (1.000 permutations); OR, odds ratio; 95%CI, 95% confidence interval; TBA, testing-balanced accuracy; S_e_, model sensitivity; S_p_, model specificity; CVC, cross-validation consistency; all models were validated through the permutation test with adjustment for covariates. The permutation test included 1000 permutations at a 10-fold cross-validation that ensured p_perm_ < 0.001; the reproducibility of the models (CVC) after the test was 100%.

**Table 5:** The potential functionality of the UF-causal SNPs rs440837 [A/G] *ZBTB10* and rs3779195 [T/A] *BAIAP2L1* and their proxy variants (r ≥ 0.80) in organism as a whole and in the liver (SHBG synthesis place) (in silico data).

UF-Causal SNPs and Their Proxy Variants	Haploreg Data			GTE-Portal Data (eQTL, sQTL)	
	Epigenetic Modifications (Methylation/Acetylation of Histones) in the Liver Promoter/Enhancer Regions	Transcription Factors	Proteins Bound	Organism	Liver
rs440837 [A/G] *ZBTB10*	H3K4me1_Enh H3K4me3_Pro H3K27ac_Enh H3K9ac_Pro	Hlx1, Hoxa9, Smad3			
5 proxy variants of rs440837 [A/G] *ZBTB10*	H3K4me1_Enh H3K4me3_Pro H3K27ac_Enh H3K9ac_Pro	Pou1f1, Pou3f2, DMRT3, DMRT4, CDP, DMRT5, Smad3, Gfi1, Mrg1: Hoxa9, Sp4, Bbx, Nkx2, Sox, Mef2, Tgif1, CTCF, Mrg, Hoxa9, LXR, Myc, TCF11: MafG, Hlx1			
rs3779195 [T/A] *BAIAP2L1*	H3K4me1_Enh H3K4me3_Pro H3K27ac_Enh H3K9ac_Pro	Foxp1		*BAIAP2L1*, *BRI3*, *TECPR1*, *LMTK2*, *RP11-307C18.1* (eQTL) *BAIAP2L1*, *BRI3*, (sQTL)	*RP11-307C18.1*, *BRI3* (eQTL)
20 proxy variants of rs3779195 [T/A] *BAIAP2L1*	H3K4me1_Enh H3K4me3_Pro H3K27ac_Enh H3K9ac_Pro	ZNF263, Znf143, Zfp161, Zfp105, VDR, TCF12, UF1H3BETA, TCF4, TATA, SP1, STAT, SRF, Sox, Sin3Ak-20, p300, RXRA, Pou6f1, Pou3f2, Pou2f2, PLZF, Pax-6, Pax-4, Pax-2, NRSF, Nrf1, Nr2f2, Nkx3, Nkx2, NF-kappaB, Ncx, MAZR, MZF1:1-4, Myc, Mef2, MAZ, Lhx3, E2F, Lmo2-complex, KAP1, Hoxd10, Hoxa9, Hoxb13, Hoxa4, Hoxa10, HNF4, HNF1, HMGN3, GR, GATA, Foxl1, FAC1, Egr-1, EBF, DMRT4, Dbx1, CTCFL, CHD2, CEBPG, BHLHE40, BATF, BAF155, Bach2, Bach1, Ascl2, Arid3a, AP-2, AP-1	USF1, SMC3R, AD21, PRDM1, MXI1, GTF2F1, POL24H8, PU1, POL2, MAX, MAFK, CTCF, HDAC2, GABP, CMYC, CEBPB, AP-2-gamma, AP-2-alpha	*AC004967.7*, *ASNS*, *BAIAP2L1*, *BRI3*, *LMTK2*, *TECPR1*, *RP11-307C18.1*, *RP11-307C18.2*, *RP11-307C18.3*, *RP11-307C18.4*, *RP11-307C18.5*, *RP11-307C18.6*, *RP11-307C18.7*, *RP11-307C18.10*, *RP11-307C18.11* (eQTL) *BRI3*, *TECPR1*, *BAIAP2L1* (sQTL)	

H3K4me1_Enh, SNP location in the region of H3K4me1 histones marking enhancers; H3K27ac_Enh, active enhancers; H3K4me3_Pro, promoters; H3K9ac_Pro, active promoters.

## Data Availability

The data generated in the present study are available from the corresponding author upon reasonable request.

## Supplementary Materials

The following supporting information can be downloaded at: https://www.mdpi.com/article/10.3390/life15071150/s1, Table S1: The GWAS data about associations of the studied candidate gene polymorphisms with the circulating SHBG and other sex hormone concentrations; Table S2: The regulatory potential of the studied SNPs; Table S3: The allele and genotype frequencies of the studied SNPs in the uterine leiomyoma and control groups; Table S4: Genotype combinations associated with uterine leiomyoma; Table S5: The regulatory effects of the endometriosis-associated loci and SNPs in high LD (r^2^ ≥ 0.80); Table S6: The eQTL effects of the endometriosis-associated SNPs in various tissues/organs; Table S7: The eQTL values of SNPs in high LD (r^2^ ≥ 0.80) with the endometriosis-associated polymorphisms; Table S8: The sQTL effects of the endometriosis-associated SNPs in various tissues/organs; Table S9: sQTL values of SNPs in high LD (r^2^ ≥ 0.80) with the endometriosis-associated polymorphisms; Table S10: Gene set enrichment analysis of biological pathways correlated with 22 TFs and 3 proteins functionally related to UF-associated rs440837 [A/G] *ZBTB10* and their proxy 5 SNPs; Table S11: Gene set enrichment analysis of biological pathways correlated with 68 TFs, 18 regulatory proteins, and 15 proteins functionally related to the UF-associated rs3779195 [T/A] *BAIAP2L1* and their proxy 20 SNPs.

## Abbreviations

The following abbreviations are used in this manuscript:

UF	Uterine fibroids
SHBG	Sex hormone-binding globulin
SHBG_con_	Sex hormone-binding globulin concentration
SNP	Single-nucleotide polymorphism
GWAS	Genome-wide association studies
BMI	Body mass index
DNA	Deoxyribonucleic acid
MB-MDR	Model-based multifactor dimensionality reduction
MDR	Multifactor dimensionality reduction
LD	Linkage disequilibrium
TFs	Transcription factors
