# Supplementary material for: Genetic Variants Linked with the Concentration of Sex Hormone-Binding Globulin Correlate with Uterine Fibroid Risk
Source: Life (Basel). 2025 Jul 21;15(7):1150. doi: 10.3390/life15071150 (PMC12301028; doi:10.3390/life15071150)
Supplement: Supplementary file 1 [file life-15-01150-s001.zip › +27.06.25--Suppl table 1.pdf]

Supplementary Table 1. The GWAS data about associations of the studied candidate gene polymorphisms with the circulating SHBG and other sex hormone concentrations

SNP, gene	Chromosome position (hg38)	Phenotype	Association (significance) (affected allele)	Reference
rs17496332 *PRMT6*	1p13.3 (107003753)	SHBG	β=-0.028 ($p=1\times10^{-11}$) (A)	[18]
rs780093 *GCKR*	2p23.3 (27519736)	SHBG	β=-0.032 ($p=2\times10^{-16}$) (T)	[18]
rs10454142 *FOXN2*	2p16.3 (48419260)	SHBG	β=0.026 ($p=1\times10^{-7}$) (T)	[18]
rs3779195 *BAIAP2L1*	7q19.3 (98364050)	SHBG	β=-0.033 ($p=3\times10^{-8}$) (A)	[18]
		SHBG (women, pre-menopause)	β=-2.41 ($p=9\times10^{-09}$) (A)	[21]
rs440837 *ZBTB10*	8q19.13 (80549739)	SHBG	β=-0.030 ($p=3\times10^{-9}$) (A)	[18]
		SHBG (women, post-menopause)	β=1.43 ($p=1\times10^{-12}$) (G)	[21]
		SHBG (men)	β=0.57 ($p=8\times10^{-9}$) (G)	[21]
rs7910927 *JMJD1C*	10q19.3 (63379150)	SHBG	β=-0.048 ($p=6\times10^{-35}$) (T)	[18]
rs4149056 *SLCO1B1*	12p12.1 (19178615)	SHBG	β=0.029 ($p=2\times10^{-8}$) (T)	[18]
		testosterone (women)	β=0.028 ($p=5\times10^{-10}$) (C)	[22]
		SHBG (women)	β=-0.065 ($p =5\times10^{-48}$) (C)	[22]
		SHBG (women, pre-menopause)	β=-0.062 ($p=8\times10^{-11}$) (C)	[22]
		SHBG (women, post-menopause)	β=-0.079 ($p=7\times10^{-34}$) (C)	[22]
		bioavailable testosterone (women)	β=0.02 ($p=2\times10^{-16}$) (C)	[21]
		SHBG (men)	β=-1.23 ($p=7\times10^{-29}$) (C)	[21]
		SHBG (women)	β=0.030 ($p=1\times10^{-73}$) (T)	[20]
		SHBG (men)	β=0.032 ($p=6\times10^{-99}$) (T)	[20]
		total testosterone (women)	β=-0.029 ($p=1\times10^{-14}$) (T)	[20]
		bioavailable testosterone (women)	β=-0.043 ($p=3\times10^{-35}$) (T)	[20]
		total	β=0.054 ($p=1\times10^{-39}$) (T)	[20]

		testosterone (men)		
rs8023580 *PPP1R19*	15q26.2 (96165062)	SHBG	β=-0.03 ($p=8\times10^{-12}$) (T)	[18]
rs11950660 *SHBG*	17p13.1 (7618597)	SHBG	β=0.103 ($p=2\times10^{-106}$) (T)	[18]
		SHBG (women)	β=6.14 ($p=1\times10^{-300}$) (T)	[21]
		SHBG (men)	β=3.9 ($p=2\times10^{-75}$) (T)	[16]
		total testosterone (men)	β=31.8 ($p=1\times10^{-41}$) (T)	[16]
