# Supplementary material for: Genetic Variants Linked with the Concentration of Sex Hormone-Binding Globulin Correlate with Uterine Fibroid Risk
Source: Life (Basel). 2025 Jul 21;15(7):1150. doi: 10.3390/life15071150 (PMC12301028; doi:10.3390/life15071150)
Supplement: Supplementary file 1 [file life-15-01150-s001.zip › --Suppl table 3.pdf]

Supplementary Table 3. The allele and genotype frequencies of the studied SNPs in the uterine leiomyoma and control groups

Chr	SNP	Gene	Minor allele	Major allele	Minor allele frequency	Number of the studied chromosomes	Genotype distribution*	H_o	H_e	P_{HWE}
Uterine leiomyoma (n=569)										
1	rs17496332	*PRMT6*	G	A	0.352	1084	69/244/229	0.450	0.456	0.778
2	rs780093	*GCKR*	T	C	0.418	1078	92/267/180	0.495	0.487	0.724
2	rs10454142	*PPP1R21*	C	T	0.310	1044	49/226/247	0.433	0.428	0.838
7	rs3779195	*BAIAP2L1*	A	T	0.189	1070	11/180/344	0.336	0.306	0.024
8	rs440837	*ZBTB10*	G	A	0.248	1012	40/171/295	0.338	0.373	0.042
10	rs7910927	*JMJD1C*	T	G	0.474	1080	120/272/148	0.504	0.499	0.863
12	rs4149056	*SLCO1B1*	C	T	0.229	1068	25/194/315	0.363	0.353	0.540
15	rs8023580	*NR2F2*	C	T	0.284	1040	39/217/264	0.417	0.406	0.590
17	rs12150660	*SHBG*	T	G	0.251	1096	32/211/305	0.385	0.376	0.650
Control (n=973)										
1	rs17496332	*PRMT6*	G	A	0.368	1820	128/413/369	0.454	0.465	0.476
2	rs780093	*GCKR*	T	C	0.399	1862	147/449/335	0.482	0.480	0.891
2	rs10454142	*PPP1R21*	C	T	0.320	1846	97/396/430	0.429	0.435	0.705
7	rs3779195	*BAIAP2L1*	A	T	0.176	1834	37/248/632	0.270	0.290	0.052
8	rs440837	*ZBTB10*	G	A	0.238	1834	42/352/523	0.384	0.362	0.083
10	rs7910927	*JMJD1C*	T	G	0.496	1862	227/469/235	0.504	0.500	0.844
12	rs4149056	*SLCO1B1*	C	T	0.230	1768	48/310/526	0.351	0.354	0.776
15	rs8023580	*NR2F2*	C	T	0.285	1862	88/355/488	0.381	0.408	0.053
17	rs12150660	*SHBG*	T	G	0.251	1876	67/336/535	0.358	0.376	0.164

Note: * minor allele homozygotes / heterozygotes / major allele homozygotes
