# Supplementary material for: Genetic Variants Linked with the Concentration of Sex Hormone-Binding Globulin Correlate with Uterine Fibroid Risk
Source: Life (Basel). 2025 Jul 21;15(7):1150. doi: 10.3390/life15071150 (PMC12301028; doi:10.3390/life15071150)
Supplement: Supplementary file 1 [file life-15-01150-s001.zip › --Suppl table 4.pdf]

Supplementary Table 4. Genotype combinations associated with uterine leiomyoma *

Model	N	Genotype combinations	*beta*	P	Risk, High/ Low
Three-order interaction models					
1	1	rs8023580-TT *NR2F2*-rs7910927-GG *JMJD1C*- rs3779195-TA *BAIAP2L1*	0.553	0.050	H
	2	rs8023580-TC *NR2F2*-rs7910927-GT *JMJD1C*- rs3779195-TA *BAIAP2L1*	0.669	0.007	H
	3	rs8023580-TT *NR2F2*-rs7910927-TT *JMJD1C*- rs3779195-TA *BAIAP2L1*	0.821	0.012	H
2	1	rs8023580-TT *NR2F2*-rs10454142-TC *PPP1R21*-rs780093-TT *GCKR*	0.934	0.006	H
	2	rs8023580-TT *NR2F2*-rs10454142-TC *PPP1R21*-rs780093-CT *GCKR*	-0.432	0.033	L
Four-order interaction models					
3	1	rs440837-AA *ZBTB10*-rs10454142-TC *PPP1R21*- rs780093-CC *GCKR*-rs17496332-AA *PRMT6*	-0.931	0.020	L
	2	rs440837-AA *ZBTB10*- rs10454142-CC *PPP1R21*-rs780093-CC *GCKR*-rs17496332-AA *PRMT6*	1.507	0.024	H
	3	rs440837-AA *ZBTB10*- rs10454142-TC *PPP1R21*-rs780093-TT *GCKR*-rs17496332-AA *PRMT6*	1.209	0.010	H
	4	rs440837-AG *ZBTB10*- rs10454142-TC *PPP1R21*-rs780093-CT *GCKR*-rs17496332-AG *PRMT6*	-0.721	0.041	L
	5	rs440837-GG *ZBTB10*- rs10454142-TC *PPP1R21*-rs780093-CT *GCKR*-rs17496332-AG *PRMT6*	2.301	0.044	H
	6	rs440837-AA *ZBTB10*- rs10454142-TC *PPP1R21*-rs780093-TT *GCKR*-rs17496332-AG *PRMT6*	0.743	0.048	H
	7	rs440837-AA *ZBTB10*- rs10454142-TT *PPP1R21*-rs780093-CT *GCKR*-rs17496332-GG *PRMT6*	0.841	0.036	H
4	1	rs8023580-TC *NR2F2*- rs10454142-CC *PPP1R21*-rs780093-CC *GCKR*-rs17496332-AA *PRMT6*	1.344	0.024	H
	2	rs8023580-TC *NR2F2*- rs10454142-CC *PPP1R21*- rs780093-CT *GCKR*- rs17496332-AA *PRMT6*	1.348	0.028	H
	3	rs8023580-TT *NR2F2*- rs10454142-TC *PPP1R21*- rs780093-TT *GCKR*- rs17496332-AA *PRMT6*	2.027	0.0007	H
	4	rs8023580-TC *NR2F2*- rs10454142-TC *PPP1R21*- rs780093-TT *GCKR*- rs17496332-AG *PRMT6*	0.935	0.038	H

* Genotype combinations are derived from the interaction models obtained by the MB-MDR method and described in Table 1.
